# Refractory Statin-Induced Immune-Mediated Necrotizing Myositis: Challenges and Perils in Its Management

**DOI:** 10.7759/cureus.24778

**Published:** 2022-05-06

**Authors:** Chong Hsien Yeo, Aziman Yaakub, Margaret Choon Lee Wang, Sylvester Andrew Shim, Pui L Chong, Muhammad Abdul Mabood Khalil, Pemasiri U Telisinghe, Kian C Lim, Jackson Tan, Vui H Chong

**Affiliations:** 1 Department of Medicine, Raja Isteri Pengiran Anak Saleha Hospital, Bandar Seri Begawan, BRN; 2 Department of Renal Medicine, Raja Isteri Pengiran Anak Saleha Hospital, Bandar Seri Begawan, BRN; 3 Department of Pathology, Raja Isteri Pengiran Anak Saleha Hospital, Bandar Seri Begawan, BRN; 4 Department of Radiology, Raja Isteri Pengiran Anak Saleha Hospital, Bandar Seri Begawan, BRN; 5 Department of Medicine, Pengiran Muda Mahkota Pengiran Muda Haji Al-Muhtadee Billah Hospital, Tutong, BRN

**Keywords:** statin-induced necrotizing autoimmune myopathy, complications, hmg-coa reductase inhibitors, myopathy, myositis

## Abstract

Statin or 3-hydroxy-3-methylglutaryl-coenzyme A reductase (HMGCR) inhibitor is widely used and plays a vital role in the management of cardiovascular and cerebrovascular diseases. Statin is generally safe and its side effects are mostly mild and self-limiting. Immune-mediated necrotizing myositis (IMNM) is a rare and serious side effect characterized by the presence of anti-HMGCR inhibitor and myositis. Long-term immunosuppressive therapy is often required to manage it, and in refractory cases, the treatment can be very challenging. We report the case of a 55-year-old female with underlying diabetes mellitus and hyperlipidemia who developed refractory statin-induced IMNM despite being administered prednisolone, methotrexate, azathioprine, and immunoglobulin. After the introduction of rituximab, steroids were able to be tapered down to the lowest maintenance dose. Unfortunately, the patient subsequently succumbed to severe coronary artery disease (CAD) likely caused by the long-term steroid therapy, highlighting the difficulty and complications associated with the treatment of IMNM, especially in patients with cardiovascular risk factors.

## Introduction

Statin or 3-hydroxy-3-methylglutaryl-coenzyme A reductase (HMGCR) inhibitor is a widely used class of medication and is the cornerstone in the management of cardiovascular and cerebrovascular diseases. It is generally considered to be very safe but can sometimes lead to serious adverse effects [1,2]. Statin-induced immune-mediated necrotizing myositis (IMNM) is a rare but increasingly recognized complication [3-6]. Antibody to the HMGCR (anti-HMGCR) is the hallmark of this condition, and patients typically present with myositis and markedly elevated serum creatine kinase (CK). Immunosuppression is often required and steroids are often the first-line treatment [5,6]. The long-term use of immunosuppression is, however, fraught with complications. Patients being refractory to treatment poses a major challenge. Given the large number of patients being treated with statins, it is important for clinicians to be aware of this rare complication and the complications associated with its treatment.

## Case presentation

A 55-year-old female was referred for the evaluation of an abnormal liver profile: alanine aminotransferase (ALT) of 251 U/L [normal range (NR): 1-54], gamma-glutamyl transferase (GGT) of 111 U/L (NR: 9-36) with normal bilirubin, alkaline phosphatase, and total protein. Hepatitis B and C markers were negative whereas anti-HAV IgG was positive, indicating past exposure. She had diabetes mellitus and hyperlipidemia, which had been diagnosed three years previously (December 2015), under the care of her community health clinic. She did not smoke or drink and had no family history of coronary artery disease (CAD). She was initiated on once-daily metformin 500 mg, gliclazide 40 mg, and atorvastatin 10 mg. After several months, atorvastatin was replaced with simvastatin (10 mg daily) by her primary care physician (PCP) on account of her arthralgia/myalgia. Her symptoms improved, but simvastatin was later stopped due to her abnormal liver profile, which led to the referral (September 2018).

At the consultation, she complained of a two-month history of myalgia and intermittent proximal muscle weakness, evident when using the stairs. On examination, she had a normal habitus (weight: 58.6 kg, height: 156.2 cm; body mass index: 24.01 kg/m^2^) and had no stigmata of chronic liver, autoimmune disease, thyroid, or Cushing disease. However, there was proximal muscle weakness (grades 4/5), mainly in the lower limbs, with difficulty in rising from sitting or squatting to standing positions. The rest of the physical examination including the neurological examination was normal.

**Figure 1:**
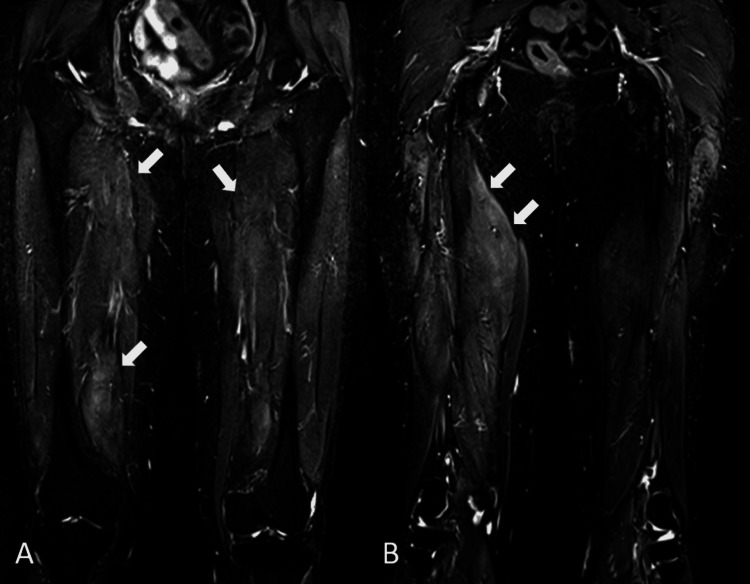
MRI of the thighs Coronal T2 TIRM sequence images through the anterior (A) and posterior (B) thighs show hyperintense signals (indicated by arrows) in the thigh muscles on both sides consistent with myositis MRI: magnetic resonance imaging; TIRM: turbo inversion recovery magnitude

Autoimmune workups (antinuclear antibody, extractable nuclear antibodies, and double-stranded DNA) were negative. Thyroid function profile and erythrocyte sedimentation rate were normal. Serum CK was markedly elevated at 6,981 U/L (NR: <300). She was referred to the rheumatology service for evaluation. Inflammatory myositis was suspected and prednisolone 20 mg daily, vitamin D, and calcium replacement were started. Due to her history of weight loss and mildly elevated carcinoembryonic antigen (5.1 ng/ml, NR: <4.9), upper and lower gastrointestinal endoscopies and CT scans (thorax, abdomen, and pelvis) were done to exclude paraneoplastic manifestations. All were normal. In light of her history of previous statin use, an anti-HMGCR antibody check was done and was found markedly elevated at >200 U/ml (NR: <20), confirming the diagnosis of statin-induced IMNM. An MRI of her thighs showed swollen muscle consistent with myositis (Figures 1A, 1B).

**Figure 2:**
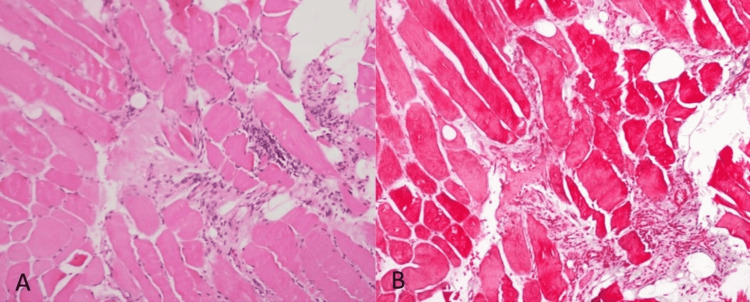
Muscle biopsies Sections were taken in transverse and longitudinal directions. There is focal atrophy of muscle fibers. A few muscle fibers are necrotic and show moderate mononuclear cell and histiocytic infiltration. Some of the muscle fibers show a moth-eaten appearance. The appearances are those of polymyositis

A muscle biopsy of the thigh showed focal atrophy, necrosis, and inflammation with minimal infiltrate (Figures 2A, 2B).

**Figure 3:**
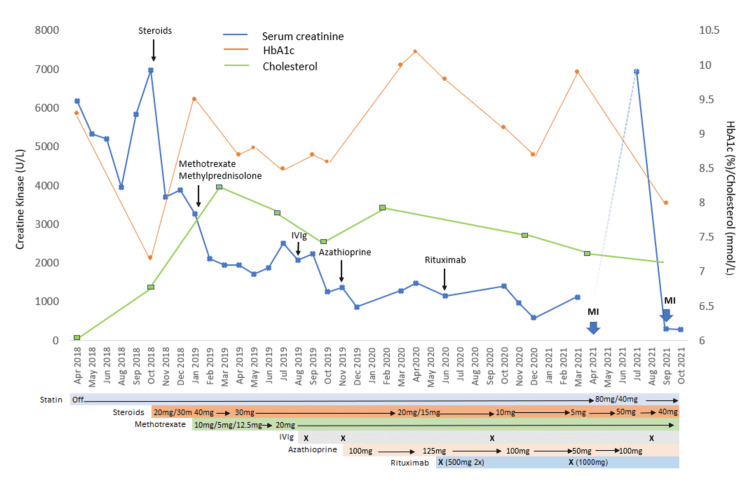
Chart showing trend of creatine kinase and events over time (introduction and use of various medications to control the myositis) Black arrows indicate the time of the initiation of medications. The big blue arrow indicates myocardial events. Colored bars indicate medications, time of their starting, and doses. X marks the time of administration of IVIg and rituximab MI: myocardial infarction; IVIg: intravenous immunoglobulin

The prednisolone dose was increased (40 mg daily). However, mild symptoms persisted, and serum CK improved but remained high (Figure 3). Oral methotrexate (12.5 mg weekly) was initiated with a dose of methylprednisolone (500 mg), resulting in some improvement in CK, and oral prednisolone was reduced to 30 mg daily. Intravenous immunoglobulin (IVIg) 5 gm/kg was given when serum CK started to increase. Azathioprine (100 mg daily) was later added along with another dose of IVIg. Although there was clinical improvement, serum CK remained elevated despite the patient being on prednisolone (20 mg daily), azathioprine (125 mg daily), and methotrexate (20 mg weekly). It was then decided to introduce rituximab, 21 months after the initial diagnosis. After rituximab (500 mg) was given for two weeks, prednisolone and azathioprine could be weaned down to 10 mg and 100 mg respectively. After undergoing rituximab therapy, the patient developed herpes zoster (T8-9) and bacterial infections that were treated with a two-week course of acyclovir and several courses of antibiotics. Although there was clinical improvement and steroids and azathioprine could be weaned down, serum CK remained elevated and a third dose of IVIg was given, resulting in a significant drop in serum CK, which enabled steroids and azathioprine to be further reduced to 5 mg and 50 mg respectively. To consolidate this, another course of rituximab was planned but was declined by the patient due to side effects (severe body aches) from a previous infusion. Nine months after rituximab, her symptoms worsened and serum CK increased, and another course of rituximab was scheduled. She received the first of two planned doses (1000 mg three weeks apart). Figure 3 shows the trends of serum CK, HbA1c, lipids, and medication use. Due to the effects of steroids, adjustments to diabetes treatment including the addition of insulin were also required.

A day after the last dose of rituximab, she presented with dizziness and nausea without chest pain or dyspnea. EKG showed inferior ST-segment elevation myocardial infarction. Serum troponin I was markedly elevated at 11,898.8 ng/L (NR: <15.5). The patient was given aspirin 300 mg, ticagrelor 180 mg, and atorvastatin 80 mg and was transferred to the cardiac center. Coronary angiogram showed severe triple vessel disease: proximal mLAD with 90% stenosis, distal left anterior descending (dLAD) artery with 100% stenosis without collaterals, and 100% stenosis of the right coronary artery (RCA). The proximal RCA stenosis was treated with percutaneous coronary intervention. However, the distal disease was deemed to be severe and not ideal for revascularization surgery. She was started on intensive medical therapy that consisted of dual antiplatelets (aspirin 100 mg daily and clopidogrel 75 mg daily), lipid (atorvastatin 40 mg daily), and glycemic and blood pressure control. Echocardiography showed a reduced ejection fraction of 40%. She eventually recovered from this event and was discharged with instructions to continue with all her medications.

When she was seen in the rheumatology clinic (July 2021), she again had a recurrence of weakness, and her serum CK was elevated (6,942 U/ml). Prednisolone was increased to 50 mg and azathioprine to 100 mg once daily. She again presented two months later to the emergency department with non-ST elevation myocardial infarction. Echocardiography showed global hypokinesia, thinned-out septum, and ejection fraction of 40%. A diagnosis of cardiac failure secondary to ischemic cardiomyopathy complicated by non-ST myocardial infarction was made. Unfortunately, her progress was complicated by basal ganglia infarction with hemorrhagic transformation and *Escherichia coli* sepsis. Despite intensive treatment, her condition deteriorated, necessitating mechanical ventilation. Soon after this, she succumbed to a cardiovascular asystolic event. The cause of death was attributed to multiorgan failure secondary to sepsis in the setting of severe CAD and refractory statin-induced IMNM. No autopsy was requested.

## Discussion

Statin-induced myopathies are categorized into self-limiting myopathy, rhabdomyolysis, and IMNM [3-6]. Myalgia, generally defined as muscle symptoms without significant CK elevation, is the most common reported side effect with approximately 1.5-5.0% of statin users mentioned in randomized controlled trials and a much higher number treated in the usual care settings with rates of 9-20% in outpatient settings [7]. Statin-induced rhabdomyolysis and IMNM are rare entities. Table 1 shows the characteristics of statin-induced myopathies [3,6,8-10].

**Table 1:** Comparison of various musculoskeletal side effects of statin therapy** **[3,6,8-10] IMNM: immune-mediated necrotizing myopathy; CK: creatine kinase; SNP: single nucleotide polymorphism; HMGCR: 3-hydroxy-3-methylglutaryl-CoA reductase; MRI: magnetic resonance imaging

	Self-limiting statin myopathy (myalgia/non-immune myositis) [6]	Rhabdomyolysis [8]	Statin-induced IMNM [3,6,9,10]
Incidence	7–29%	0.3–13.5/1,000,000 users (0.0003–0.0013%)	2–3/100,000 users (0.002–0.003%)
Age range	Increased risk with age	Median age: 64 (SD: 14) years. Increased risk with age	Increased risk with older age. Median age: 64 (range: 52-86 years)
Gender predisposition	Female	Male (70%)	Female (two-thirds of those affected)
Dose and interval	Dose-related	Dose-related	Any time and can be after stopping therapy
Risks	Increasing age (>80 years), female sex, polypharmacy, comorbidities (hypothyroid, diabetes, hypertension, muscle metabolic disease, and renal disease), reduced muscle mass, and impaired renal function	Increasing age (>80 years), female sex, polypharmacy, comorbidities (hypothyroid, diabetes, hypertension, muscle metabolic disease, and renal disease), reduced muscle mass, and impaired renal function	Increasing age (>80 years), female sex, polypharmacy, comorbidities (hypothyroid, diabetes, hypertension, muscle metabolic disease, and renal disease), reduced muscle mass, and impaired renal function
Manifestation			
Myalgia	Common	Common and painful. Associated with myoglobinuria. Acute renal failure	Common
Proximal muscle weakness	Uncommon	Common. Non-specific	Common. Upper and lower limbs
Genetic risk factor	SNP in SLC01B1	SNP in SLC01B1	Anti-HMGCR HLA-DRB1*11: 01 and 07: 01
Diagnosis	Clinical muscle biopsy not required	Marked elevation of CK. Muscle biopsy not required	Positive (anti-HMGCR), myositis (elevated CK), and symptoms. Muscle biopsy not required for diagnosis
Muscle biopsy	Variable findings	Muscle necrosis	Necrosis, degeneration, regeneration, fibrosis, and pauci-inflammatory cells
MRI	Normal may show muscle edema	Diffuse swelling of affected muscle groups. Areas of breakdown with liquefied necrosis	Muscle edema
Management	Withdrawal of statin	Withdrawal of statin	Withdrawal of statin
		Hydration	Immunosuppressive therapy
Outcome	Self-limiting	Self-limiting	Subacute in 2/3 and progressive in 1/3

Statin-induced IMNM is a subset of IMNM; the European Neuromuscular Center criteria recognize three distinct subtypes: anti-signal recognition particle (anti-SRP) myopathy (~70%), anti-HMGCR myopathy (20%), and autoantibody-negative (negatives for anti-SRP and anti-HMGCR) IMNM (<10%) [11-13]. Apart from being specific with respect to subtypes, antibody levels also correlate with disease activities. Anti-SRP myopathy is associated with more severe muscle involvement, has more common extra-muscular features, and may respond better to therapy including rituximab compared to anti-HMGCR. Very rarely, a patient can be positive for both these myopathic-specific antibodies [14]. Patients in the third group may be positive for other autoimmune antibodies, indicating an association with other autoimmune diseases [14], but will need to be evaluated for other causes such as infectious diseases (HIV or HCV), malignancies, and toxins such as those related to medications [11,14].

The reported incidence of statin-induced IMNM varies depending on the region. However, it is estimated that approximately 1-3/100,000/year statin users are affected [3,9], with the risk increasing with age, and women being affected (~two-thirds) more than men [3,4-6,9,12]. The underlying pathogenesis is believed to trigger the complement inflammatory response by the anti-HMGCR antibody [2,3]. Statin treatment blocks the metabolic pathway of cholesterol and leads to an increase in the expression of HMGCR, and in susceptible persons, results in the development of anti-HMGCR. It is a class effect but remains uncertain if it is dose-dependent, and can occur even a long time after stopping the statin. However, it has been shown that atorvastatin, lovastatin, and simvastatin (metabolized by cytochrome P450 3A4) were associated with higher rates of adverse effects (4.2 per 100,000 person-years) compared to pravastatin and fluvastatin (not metabolized by CP450 3A4) [15]. Diagnosis requires the presence of elevated CK and anti-HMGCR antibodies [2,3,5,6]. Muscle biopsy is not required for diagnosis, but patients typically show muscle edema, atrophy, and pauci-inflammatory infiltrates.

In most cases, symptoms are present at diagnosis and typically manifest with proximal muscle weakness: lower and upper. Proximal upper limb-only weakness can be confused with shoulder girdle myopathy. Manifestation with dysphagia has also been reported [16]. If untreated, the symptoms become more severe with resultant muscle loss and weakness. Even with therapy, patients may not recover completely in part due to the disease and the side effects of steroid therapy, which is associated with myopathy.

The management can be challenging due to a lack of treatment guidelines, particularly for refractory cases. Generally, management requires statin discontinuation and initiation of immunosuppressive therapy, which should be started early [3-6,9,12]. Prednisolone is regarded as the first line of therapy, followed by steroid-sparing agents such as methotrexate, azathioprine, mycophenolate mofetil, IVIg, or rituximab [3-6,9,12]. Interestingly, there has been a report of a case with only persistent hyper CK but no symptoms [17]. Despite stepwise improvements with the addition of immunosuppressants, remission could not be achieved in our patient. Although steroid dosage could be reduced, it was only after the introduction of rituximab and an additional dose of IVIg that further reduction to the lowest steroids and azathioprine doses was achieved (Figure 3). Rituximab, a potent B-cell-depleting agent has been shown to be effective in steroid-refractory IMNM, especially anti-SRP and statin-naive IMNM. Several studies have shown that the early introduction was successful in inducing remissions in refractory cases [18-20]. However, the introduction of rituximab was complicated by herpes zosters and bacterial infections, highlighting the well-known and feared risks associated with this treatment. Our case highlights the challenges encountered in the management of refractory IMNM. Others have recommended an early initiation of triple therapy, steroids, IVIg, and steroid-sparing agents, which has been shown to preserve muscle strength [21]. In some anti-HMGCR patients, IVIg may be effective, even as monotherapy [12]. The use of plasma exchange and therapy targeting the complement pathway has been proposed due to the pathogenic effects of anti-HMGCR and anti-SRP antibodies on muscles in an in vivo study [22].

Approximately two-thirds of cases run a subacute and the rest run a progressive course [6]. Prognosis is reported to be better with older age at diagnosis and early interventions [6,23,24]. It has been reported that the majority of patients above 60 years recovered full strength (85%) within four years, compared to less than half of the patients who were below 52 years of age [23]. Our patient had the progressive type, and even though it was eventually controlled, she, unfortunately, had accrued a long period of high-dose steroid exposure, which likely contributed to her severe CAD.

There are several aspects of our case that need to be highlighted. Firstly, the long-term use of high-dose steroids should generally be avoided with the early introduction of steroid-sparing agents. The long-term use of prednisolone likely contributed to the development of severe CAD. Apart from diabetes and dyslipidemia, our patient did not have any other risk factors. In retrospect, a coronary assessment (i.e., CT calcium score or CT coronary angiogram) would have been useful in stratifying her cardiac risk. Secondly, the early introduction of rituximab should have been considered and might have altered the outcome. Unfortunately, we did not consider it due to a lack of evidence and treatment recommendations, and a high risk of sepsis complications. Recently, several studies have reported the effectiveness of rituximab in refractory IMNM [18-20]. Third, It is pertinent to consider if rituximab was associated with the myocardial event in our case? Rituximab-induced myocardial events have been reported and are believed to be due to hypersensitivity reactions [25]. Our patient had severe CAD and even a minor reaction would be detrimental. Fourth, IMNM is a rare entity, and many clinicians are unaware of this condition and its association with statins, even clinicians who commonly use statins. Our patient was restarted on high-dose statin following the first myocardial infarction as per standard management guidelines. She also did not complain of muscle symptoms until several months after restarting treatments, indicating that relapse does not necessarily occur immediately after re-exposure to statin. Finally, long-term immunosuppressive therapy is fraught with infective complications, a well-known fact that can be overlooked in modern-day practices, especially with an increasing number of patients being treated. Even though immunosuppressive treatments were stopped during our patient's last illness, the long-term effects of immunosuppressants may have contributed to her septic complications.

## Conclusions

Our report described an interesting case of refractory statin-induced IMNM and highlighted the challenges faced in its management. The challenges in management arise not just from the condition itself but also from complications that result from the treatment. With the rising incidence of metabolic disorders, less common and rare complications such as statin-induced IMNM are expected to increase. It is important to be aware of this rare yet serious complication of statins. Equally important is the awareness of the complications due to immunosuppressive therapy required to treat this condition. Early introduction of IVIg and rituximab should be considered in the treatment of steroid-refractory IMNM.
